# A non-weight bearing protocol after ACL reconstruction improves static anterior tibial translation in patients with elevated slope and increased weight bearing tibial anterior translation

**DOI:** 10.1186/s40634-023-00694-w

**Published:** 2023-12-20

**Authors:** Iacopo Romandini, Nicolas Cance, Michael J. Dan, Tomas Pineda, Benoit Pairot de Fontenay, Guillaume Demey, David H. Dejour

**Affiliations:** 1 Orthopedic Surgery Department, Lyon Ortho Clinic, Clinique de La Sauvegarde, 29 Avenue Des Sources, 69009 Lyon, France; 2 IRCCS Istituto Ortopedico Rizzoli, Clinica Ortopedica e Traumatologica 2, Via Pupilli 1, Bologna, 40136 Italy; 3 Surgical and Orthopaedic Research Laboratory, Prince of Wales Clinical School, University of New South Wales, 2052 Sydney, Australia; 4 Hospital El Carmen, Santiago, Chile

**Keywords:** Anterior cruciate ligament, Weight bearing, Arthroscopy, Knee, Post-operative protocol

## Abstract

**Purpose:**

Aim of this study is to evaluate the impact of a non-weight bearing (NWB) protocol within 21 post-operative days after anterior cruciate ligament (ACL) reconstruction on static and dynamic anterior tibial translations (SATT and DATT, respectively). The hypothesis is that delayed WB would improve ATT at 9 months follow-up.

**Methods:**

A series of patients treated with ACL reconstruction was retrospectively reviewed, comparing a group with immediate post-operative weight bearing (WB group) and a group without post-operative weight bearing (NWB group). The NWB protocol was applied to patients with posterior tibial slope (PTS) ≥ 12°, pre-operative SATT ≥ 5 mm, and/or meniscal lesions of root or radial type. SATT, and PTS were measured on 20° flexion monopodal lateral x-rays, while DATT on Telos™ x-rays at pre-operative and 9-months follow-up.

**Results:**

One hundred seventy-nine patients were included (50 NWB group, 129 WB group). The SATT worsened in the WB group with a mean increase of 0.7 mm (SD 3.1 mm), while in the NWB group, the SATT improved with a mean decrease of 1.4 mm (SD 3.1 mm) from the pre-operative to 9 months’ follow-up (*p* < 0.001). The side-to-side Telos™ evaluation showed a significant improvement in DATT within both the groups (*p* < 0.001), but there was no difference between the two groups (*p* = 0.99).

**Conclusion:**

The post-operative protocol of 21 days without WB led to an improvement in SATT at 9 months without an influence on DATT, and it is recommended for patients with a SATT ≥ 5 mm and/or a PTS ≥ 12° as part of an “à la carte” approach to ACL reconstruction.

**Level of evidence:**

Level IV, Retrospective case series

## Introduction

The anterior cruciate ligament (ACL) is the primary restraint to anterior translation and contributes to internal rotation [1] control with the anterolateral ligamentous complex. ACL ruptures are common, with increased incidence in the young, who engage in sports involving jumping and pivoting. Various intrinsic and extrinsic risk factors of rupture have been identified for ACL tear, these include anatomical variations, neuromuscular deficits, biomechanical abnormalities, playing environment, and hormonal status [2].

ACL injury increases anterior translation of the tibia relative to the femur [3]. Among the anatomical characteristics, the posterior tibial slope (PTS) and meniscal tears have been demonstrated to increase the anterior tibial translation in ACL injury (ATT) [4, 5]. On monopodal weight bearing (WB) x-rays this translation is termed static anterior tibial translation (SATT).

The degree of PTS can be measured on lateral view radiographs, while anteroposterior and rotational laxity can be subjectively evaluated using manual tests such as the Lachman and Pivot-shift tests [6], and objectively by using instruments such as the KT-1000, GNRB®, accelerometer (KiRA), Rolimeter™, or Telos™ devices [7–10]. These assessments aid surgeons by quantifying preoperative laxity, helping to guide treatment decisions (such as PTS correction or meniscal injury repair), and objectively evaluating success of the reconstruction by measuring postoperative laxity and residual ATT [11]. Residual laxity has been shown as proof of elongation cause of the excessive stress on the graft. However, the factors influencing postoperative laxity are not yet fully understood. One of the debated factors is post-operative weight-bearing (WB) [12].

Successful rehabilitation outcomes have been consistently achieved with the principle of early WB for isolated ACL procedures [13–17]. However, when combined with meniscal repair, cartilage treatments, or correction of malalignment, WB protocols may need to be adjusted to partial or NWB [13, 18, 19]. These criteria should also consider objective data on preoperative laxity, including PTS measurements and the type of meniscal injury, as these factors could contribute to increased stress on the reconstructed ACL [5, 20]. Recent studies have highlighted the influence of PTS on both static (SATT) and dynamic anterior tibial translation (DATT) following ACLR, particularly when combined with meniscal procedures [5, 20–23], while the cut-off to which those parameters influence the translation remains unclear [21, 24]. In light of factors increasing post-operative laxity, NWB may be beneficial during graft incorporation.

The principle aim of the present study was to evaluate the effects of a 21-day NWB rehabilitation protocol after ACLR on SATT and DATT. The hypothesis is that a non-weight bearing rehabilitation protocol in selected patients based on a high degree of PTS, significant SATT, and the presence of preoperative meniscal lesions could improve both SATT and DATT at the 9-month follow-up evaluation.

## Materials and methods

### Patients’ selection

The study was approved by the local ethical board (**blind number**) and all patients provided informed consent for the use of their data for research. All consecutive ACLR were performed by a single senior author (D. H. D.) between January 2017 and December 2021 were reviewed.

Inclusion criteria were: male and female patients aged over 15 years old, undergoing a primary ACLR using hamstring autograft and a minimum follow-up of 9 months. Patients were required to have adequate pre-operative and post-operative x-rays for adequate assessment of SATT, DATT and PTS. Exclusion criteria included ACL revision, major concurrent procedures such as extra-articular tenodesis (modified Lemaire), osteotomy, and absence of appropriate pre-operative or post-operative x-ray assessment. All post-operative WB protocols were available in the surgery report. Patients were then divided into 2 groups based on the following rehabilitation criteria: WB protocol (WB group) and non-WB protocol (NWB group).

### Images and measurement

Preoperative and postoperative images at 9 months follow-up were evaluated. Lateral monopodal weight-bearing knee x-rays were performed at 20º of knee flexion. Dynamic radiographs were performed, using the Telos™ stress radiography device (Metax, Hungen, Germany), and applying a constant anterior force of 150N.

**Fig. 1:**
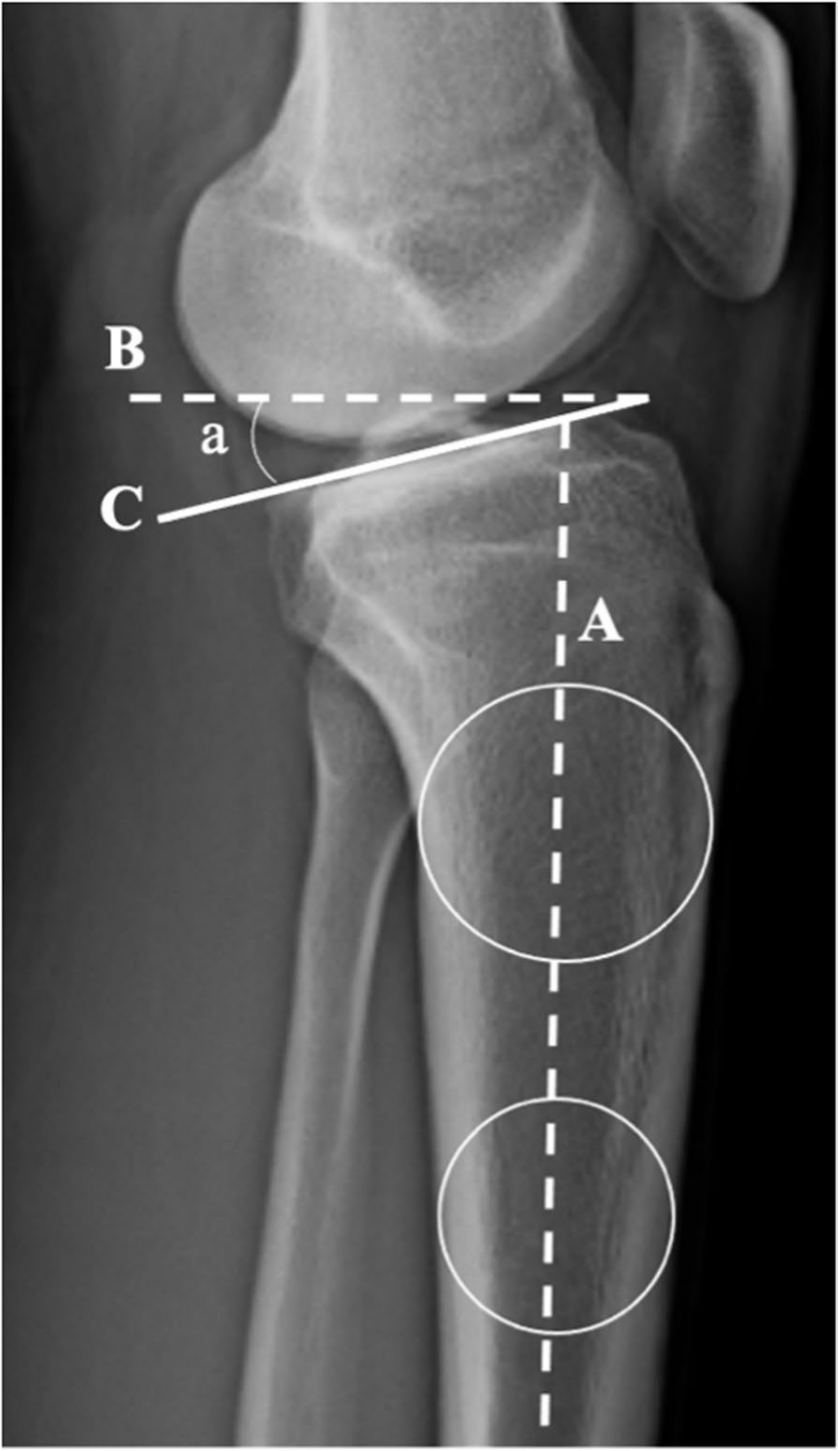
Lateral right knee radiograph demonstrating Posterior Tibial Slope (PTS). Measurement of PTS in monopodal WB x-rays. PTS is the angle formed between a line (**B**) perpendicular to the tibial diaphyseal axis (**A**) and the line (**C**) tangent to the most superior points at the anterior and posterior edges of the medial plateau

**Fig. 2:**
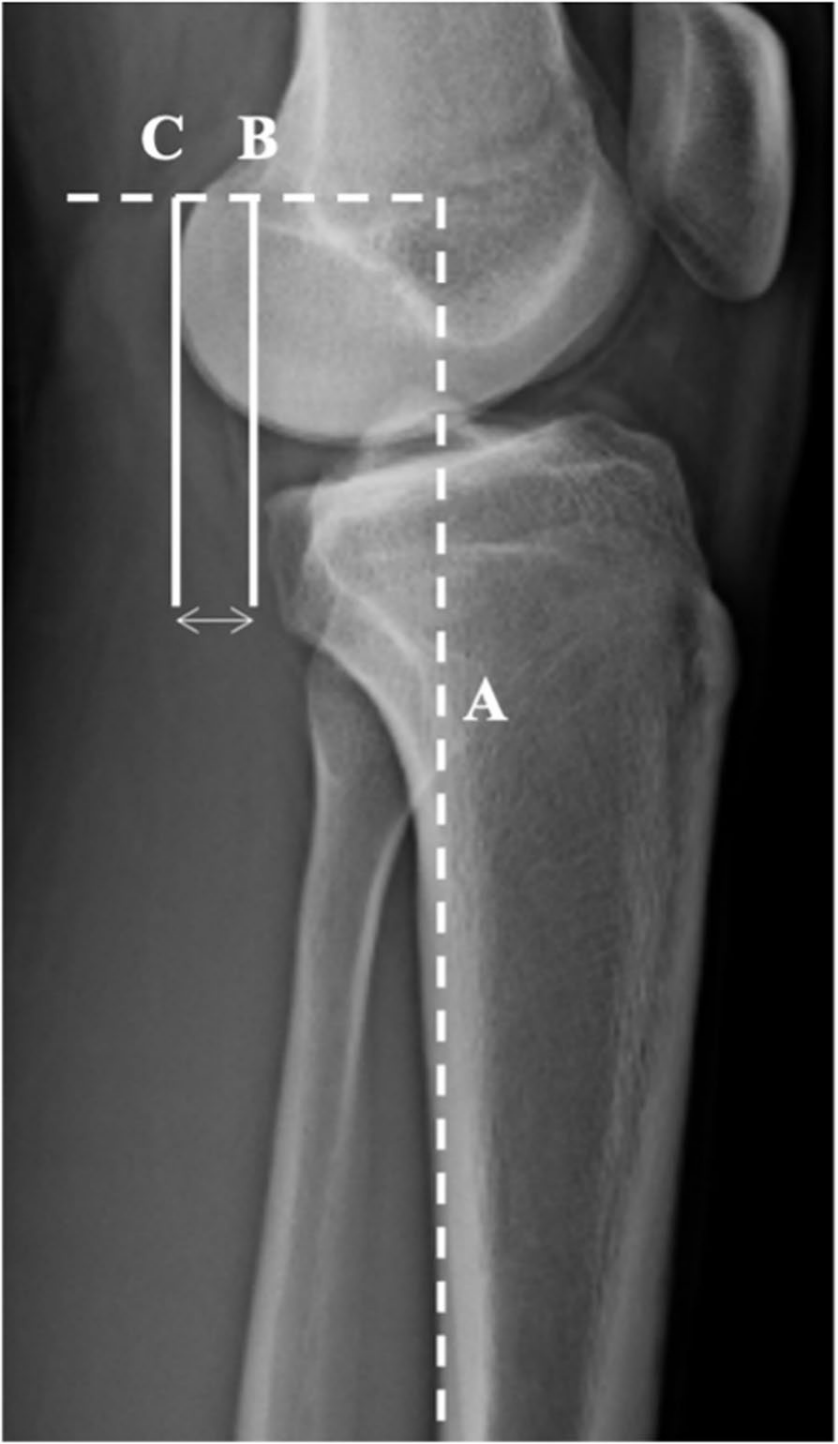
Lateral right knee radiograph demonstrating Static Anterior Tibial Translation (SATT). Measurement of SATT in monopodal WB x-rays. The posterior tibial cortex is the reference (line **A**). Two lines are traced parallel to line A and. tangent to posterior part of the medial plateau (line **B**) and medial femoral condyle (line **C**). SATT is the distance between line **B** and **C**

**Fig. 3:**
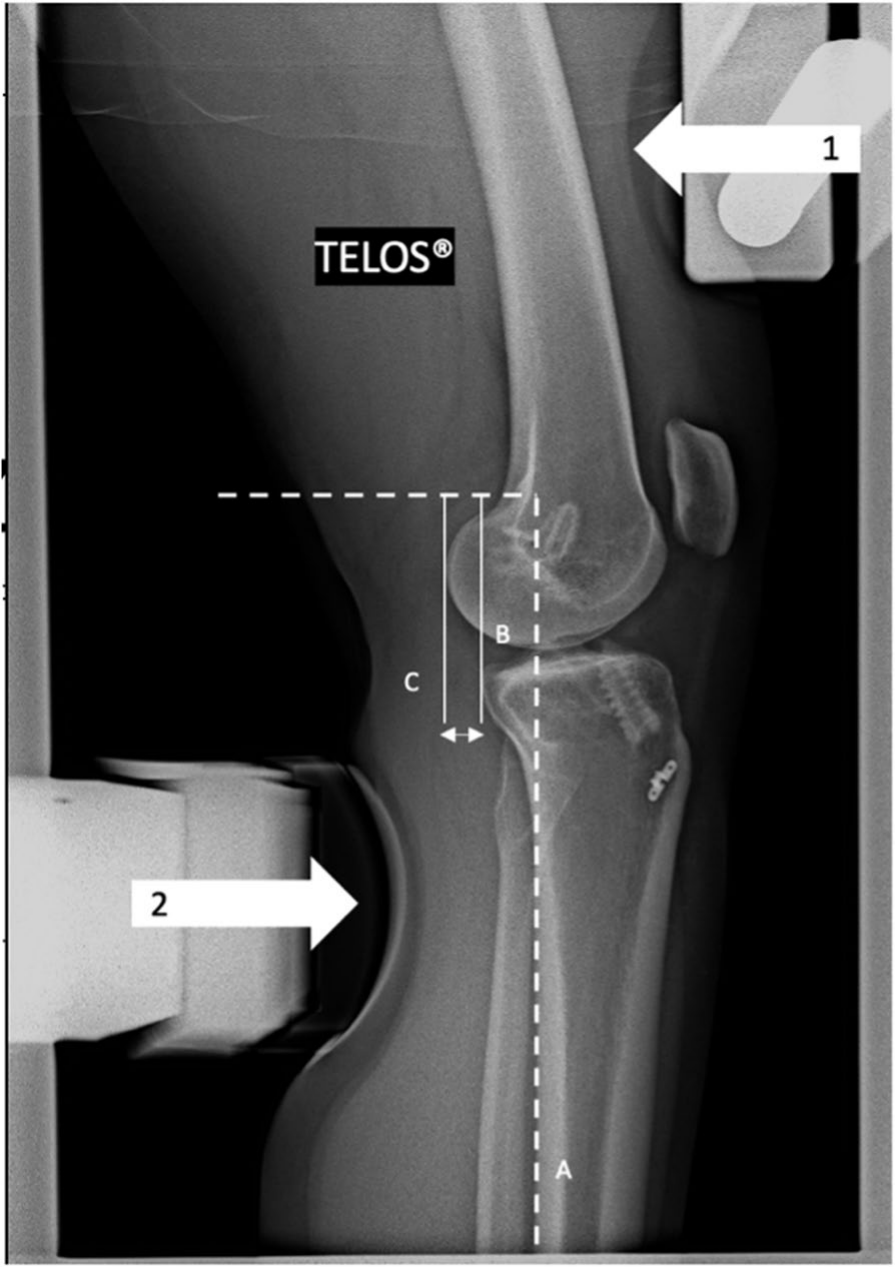
Lateral right knee radiograph demonstrating Dynamic Anterior Tibial Translation (DATT). Measurement of DATT in Telos™ procedure x-rays, using 150N force applying on the femur (arrow 1) and the tibia (arrow 2), on opposite direction. The posterior tibial cortex is the reference (line **A**). Two lines are traced parallel to line **A** and tangent to posterior part of the medial plateau (line **B**) and medial femoral condyle (line **C**). SATT is the distance between line **B** and **C**

All measurements were performed twice (1-month delay between each measurement) using Horos DICOM viewer software (version 3.3.6) on pre-operative’s x-rays and at 9 months post-operative by two independent reviewers (orthopaedic surgeons: N. C. and I. R.). The 9 months of follow-up corresponds to the last clinical and radiological evaluation before returning to pivot contact sports, and to a mid-term delay, as the final evolution of the graft and the final phase of rehabilitation. Anteroposterior knee laxity was assessed by measuring the ATT using both static and dynamic measurements on true lateral view radiographs, with the posterior femoral condyles superimposed. The ATT was determined as the distance between two parallel lines drawn on the radiograph: the first tangent to the posterior aspect of the medial tibial plateau and the second tangent to the posterior femoral condyles [24, 25]. SATT was measured on monopodal WB radiographs with the knee flexed at 20° (Fig. 1). DATT was measured using the Telos™ system, and the side-to-side difference (SSD) between the injured knee and the healthy knee was calculated (Fig. 2). The PTS was also measured on true lateral radiographs by determining the angle between the perpendicular line to the tibial diaphysis and the tangent line to the anterior and posterior edges of the medial tibial plateau [24–27] (Fig. 3). Meniscal tears were documented in the operation report.

### Surgical technique

All ACL reconstructions were performed under a general anaesthetic with a high thigh tourniquet in place. Arthroscopic examination was performed to confirm the presence of meniscal lesions and assess the ligament status through direct visualization and probing. Meniscal treatments were performed as necessary, with suture repair (Fast-Fix, Smith & Nephew, Memphis, TN) utilized for red-red or red-white lesions, and partial meniscectomy for white-white lesions. In cases of posterior root lesions, a transosseous suture repair with Number 2 Ultrabraid™ (Smith & Nephew, Memphis, TN, USA) was performed to enhance meniscal stability using a suture passer (FirstPass, Smith & Nephew, Memphis, TN). The graft used for reconstruction was obtained from the patient's autologous hamstring tendons (semitendinosus and gracilis). A four-strand configuration with two tendons [Semitendinous-Gracilis (SG)] or a single hamstring tendon [Quadrupled Semitendinous (ST4)] was created to achieve a graft diameter of 8 to 9 mm. In case of SG configuration, hamstrings were keeping attached to their tibial insertions and suturing them together to achieve the appropriate length and thickness for the patient's size. In case of QS configuration, the graft was detached from its distal attachment. The femoral tunnel was prepared using an outside-in guide, and the graft was secured using Ligafix interference screws for SG (SBM, Lourdes, France) or Pullup adjustable suspensory fixation systems (SBM, Lourdes, France).

### Postoperative rehabilitation

Following an investigation into factors affecting SATT that demonstrated no improvement in SATT following ACLR with a post operative WB protocol [21], the decision was made to implement a NWB protocol based on specific criteria to determine if this would improve SATT post-ACLR. The criteria for NWB post-operatively included: a PTS ≥ 12°, a pre-operative SATT measurement of ≥ 5 mm, and/or the presence of a root or radial type meniscal lesion. In the NWB rehab protocol, the patients were NWB for the 21 first days, and a gradual transition to full WB was allowed between 3- and 6-weeks post-surgery. For the WB rehab protocol, partial to full WB with crutches was allowed starting on the day of surgery. In both groups, physiotherapy used a non-aggressive rehabilitation protocol, with emphasis on avoiding hyperextension, without restricting flexion, was initiated immediately after the surgery for the first 45 days post operatively. After this initial period, all patients participated in the same rehabilitation program. Non-contact pivoting activities were allowed at 6 months, and if at 9 months isokinetic testing demonstrated quadriceps/hamstrings ratio and less than ten percent difference with side-to-side testing, the patients were able to return to full sports activities.

### Statistical analysis

A two-tailed Student’s t test for independent samples was used to compare the mean values of the difference between the SATT and DATT at pre, post operative and difference between pre and post operatively. SPSS (v25; IBM) was used to perform these statistical analyses. Significance was set at an alpha of *p* < 0.05.

## Results

**Fig. 4:**
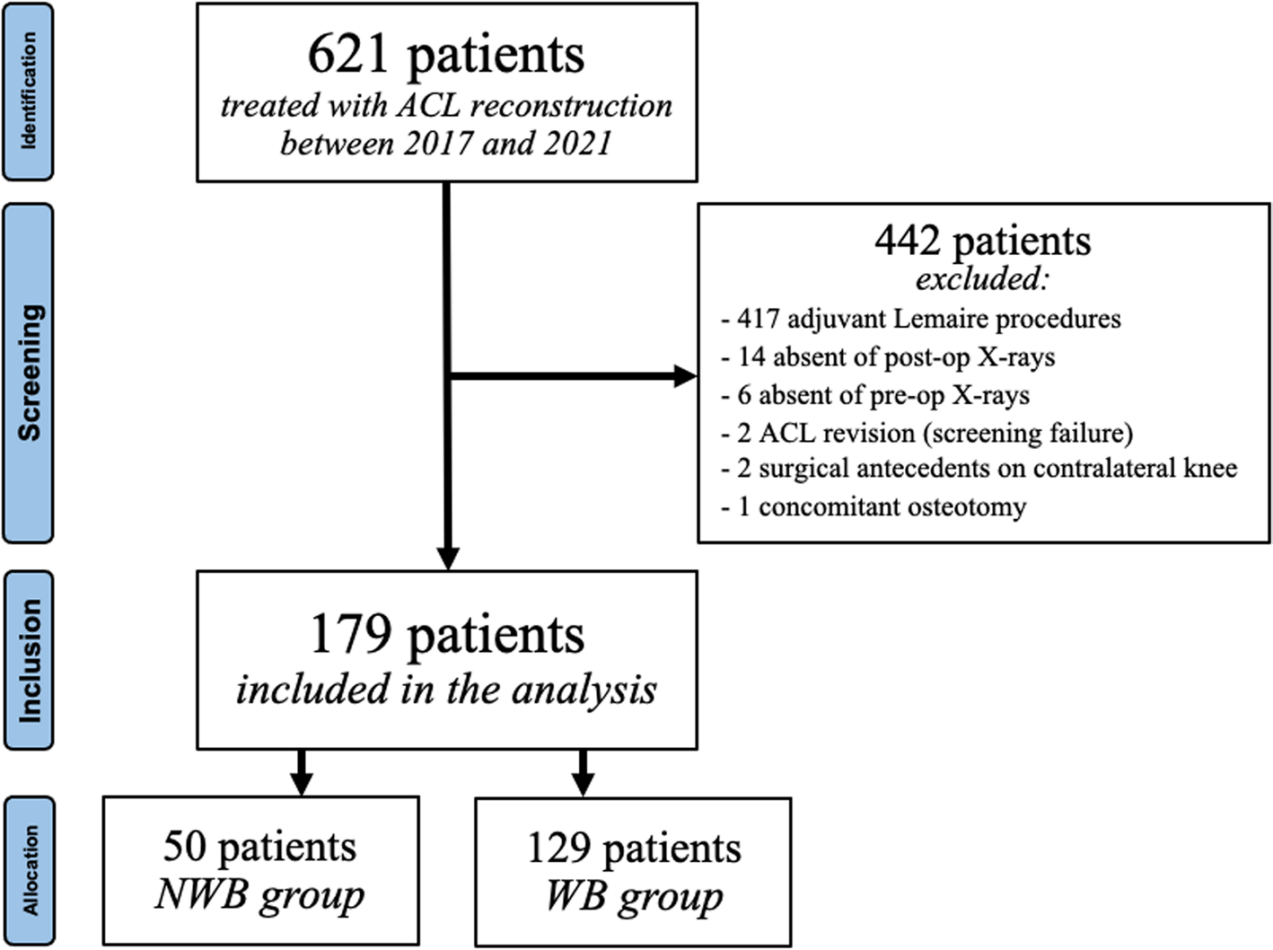
Flowchart of included patients

A total of 621 patients were treated with primary ACL reconstruction, and 179 patients were eligible and included in the analysis (Fig. 4): 50 patients (25 men and 25 women, aged 32.8 ± 11.1 years) met the criteria in the NWB group, while 129 patients (61 men and 68 women, aged 34.4 ± 11.1 years) met the criteria in the WB group. See Table 1 for surgical details.

**Table 1:** Demographics and surgical details (*)

	**Non-WB group** **(*n* = 52)**	**WB group** **(*n* = 162)**	***p-value***
Age at surgery, y	33.0 ± 10.0	33.5 ± 10.8	*n.s.*
Sex			
Male	26 (50.0)	78 (48.1)	*n.s.*
Female	26 (50.0)	84 (51.9)	*n.s.*
Knee side			
Right	25 (48.1)	86 (53.1)	*n.s.*
Left	27 (51.9)	76 (46.9)	*n.s.*
Type of ACL graft			
SG	32 (61.5)	99 (61.1)	*n.s.*
ST4	20 (38.5)	63 (38.9)	*n.s.*
Concurrent meniscal surgery			
No	11 (21.2)	82 (50.6)	*p* = *0.02*
Medial	28 (53.8)	35 (21.6)	*p* = *0.03*
Lateral	19 (36.5)	29 (17.9)	*p* = *0.05*

*y* years, *ACL* anterior cruciate ligament, *SG* Semitendinous-Gracilis, *ST4* quadrupled Semitendinous, *WB* weight bearing, *n.s.* not significant

*Data are expressed as mean ± SD (standard deviation) or number (% of patients within the group)

A total of 442 patients did not meet the inclusion criteria, as shown in Fig. 4: 417 patients (69.4%) underwent an extra-articular tenodesis (modified Lemaire), while 20 patients (3.2%) did not perform the pre-operative or 9-month follow-up radiological evaluation using the same method as illustrated in the "Images and Measurements" section. Two patients (0.3%) had undergone ACL revision, and therefore, they were excluded due to screening failure. The other 3 patients (0.4%) were not included in the analysis, according to the exclusion criteria (Fig. 4).

In the NWB group, the medial meniscal tears consisted of 19 ramp tears, 1 root tear, 1 radial tear, and 1 bucket handle tear. The lateral meniscal tears consisted of 11 root tears type 2 (according to Krych et al. [28]), 7 longitudinal tears, and 1 bucket handle tear (Table 1). In the WB group. The medial meniscal tears consisted of 22 ramp tears, 8 degenerative tears, 4 radial tears, and 1 bucket handle tears. The lateral meniscal tears consisted of 12 longitudinal tears, 13 partial radial tears, 2 horizontal tears, and 2 degenerative tears.

No significant difference was found between the 2 groups in terms of age, sex, knee side, and graft diameter or surgical technique (quadrupled vs doubled hamstring), as shown in Table 1. The analysis revealed a higher incidence of meniscal injuries (Table 1), both medial (*p* = 0.03) and lateral (*p* = 0.05), in the NWB group compared to the WB group and in patients with SATT ≥ 5 mm and PTS ≥ 12° (*p* = 0.02). In accordance with the inclusion criteria, there were a statistically significant difference between the pre-operative SATT, DTTA and PTS between the 2 groups (*p* < 0.001), as shown in Table 1.

The pre-operative mean SATT was 0.6 mm (SD 2.6 mm) in the WB group and 5.0 mm (SD 3.5 mm) in the non-WB group, and these measures were significant different (*p* < 0.001). The post-operative SATT was significantly different between the two groups with a mean of 1.4 mm (SD 3.0 mm) in the WB group and 3.9 mm (SD 3.0 mm) in the NWB group (*p* < 0.001). There was a mean increase in SATT from pre- to post-operatively in the WB group 0.7 mm (SD 3.1 mm), whereas in the NWB group there was a decrease in SATT from pre- to post-operatively of 1.4 mm (SD 3.1 mm). This was statistically significant (*p* < 0.001), as shown in Table 2.

**Table 2:** Radiological results (*)

	**non-WB group** **(*n* = 52)**	**WB group** **(*n* = 162)**	***p-value***
PTS	11.2 ± 2.5	7.9 ± 2.3	*p* < *0.001*
Pre-op SATT	5.0 ± 3.5	0.6 ± 2.6	*p* < *0.001*
Post-op SATT	3.9 ± 3.0	1.4 ± 3.0	*p* < *0.001*
Δ SATT	-1.4 ± 3.1	0.7 ± 3.1	*p* < *0.001*
Pre-op DATT	10.3 ± 3.7	7.7 ± 2.8	*p* < *0.001*
Post-op DATT	7.4 ± 3.1	4.8 ± 2.4	*p* < *0.001*
Δ DATT	2.9 ± 3.1	2.9 ± 3.0	*p* = *0.99*
STS pre-op DATT	5.0 ± 4.8	4.4 ± 3.8	*p* < *0.001*
STS post-op DATT	7.4 ± 3.1	4.9 ± 2.4	*p* < *0.001*

*PTS* posterior tibial slope, *SATT* static anterior tibial translation, *Δ* the difference between post-op and pre-op, *DATT* dynamic anterior tibial translation, *STS* the side-to-side difference between the index and contralateral knee, *WB* weight bearing

*Data are expressed as mean ± SD

The side-to-side evaluation on Telos™ radiographs showed a significant improvement in DATT within the groups (*p* < 0.001), although the difference in improvement between the two groups was not significant (*p* = 0.99): in fact, the mean difference between pre-operative and post-operative DATT in WB group were 2.9 mm (SD 3.1 mm), while the mean difference between pre-operative and post-operative DATT in NWB group were 2.9 mm (SD 3.0 mm), as shown in Table 2.

Further analysis was performed to evaluate the parameters which might influence the results. No significant correlation was found between radiological outcomes and patients’ sex, side, age, surgical technique (quadrupled vs doubled hamstrings).

A sub-analysis was conducted among patients with PTS ≥ 12° and high pre-operative laxity (SATT ≥ 5 mm), both with and without meniscal injuries. The results did not show a statistically significant difference in the reduction of SATT between patients without meniscal tears (0.5 mm, SD 2.6 mm) compared to those who had a meniscal tear (2.1 mm, SD 3.3 mm), regardless of the treatment received (*p* = 0.06). Similarly, no significant difference was found between patients without and with meniscal tears in the DATT improvement (3.28 mm, SD 3.09 mm compared to 3.35 mm, SD 3.03 mm respectively, *p* = 0.29).

No complications were recorded in either group during surgery or post-operatively. At last follow-up, no graft failure was reported. All patients returned to sport according to the rehabilitation protocol, allowing for return to contact-pivoting sports after 9 months rehab.

## Discussion

The main finding of this study is that delaying WB for up to 21 days after ACL reconstruction improves residual SATT and does not affect DATT at 9-month follow-up evaluation. These results demonstrate that the SATT and DATT [29, 30] are different and independent measures of knee laxity [31–33]. The SATT represents the load when walking during the stance phase, while dynamics tests are not suitable to individual characteristics (height or weight), and therefore differs based on the physiological stress the knee is subjected to.

The pre-operative laxity was assessed using radiographs in a pre-operative static and dynamic assessment, along with measuring PTS. The choice to use these parameters was dictated by the fact that they are easily accessible and highly reproducible measurements, in order to create individualized rehabilitation programs for patients. Moreover, some authors reported that residual knee laxity after ACLR has been strongly associated with an increased graft failure [34, 35], and the evaluation of the pre- and post-operative laxity using SATT measurement can represent an efficient technique to identify high-risk graft failure patient. Therefore, whereas the current study did not evaluate clinical outcomes as failure at more than 9 months, the decreasing of the SATT introduce a possible diminution of the failure rate using a NWB protocol in particular cases.

The importance of evaluating the post-operative WB has also been previously the subject of intense debate [12, 16, 36–38]. In recent years, there has been a consensus in favour of early WB for these patients, as numerous studies have demonstrated its positive impact on clinical outcomes, resumption of daily activities, and return to sports [15, 17, 19, 39]. Guidelines by the American Physical Therapy Association recommend early WB for patients without any negative effects on stability or function [40]. However, it is important to consider this issue in the comprehensive evaluation of patients after ACL reconstruction, as highlighted by other authors. A recent meta-analysis conducted by Fan et al. revealed a significant association between early WB in the rehabilitation program and increased anteroposterior laxity in the short-term follow-up [12]. Moreover, while the NWB rehab protocol is accepted for meniscal tears, such as radial or root, its use for preoperative laxity or any intrinsic risk factors is not widely practiced.

The importance of NWB contributing to improve SATT can be better understood by examining the process of graft ligamentization in the early weeks following surgery. The process of ligamentization involves a series of stages including inflammation, necrosis, revascularization, cellular repopulation, and synthesis of new matrix. Preclinical studies conducted on animal models have demonstrated that lower mechanical loads during the initial weeks result in reduced scar tissue formation, decreased expression of matrix metalloproteinase 13 (MMP-13), and a more organized interface between the tendon and bone [41–43]. According to Camp et al., a defined period of immobilization without WB following ACL reconstruction appears to enhance the biomechanical strength of the healing tendon-bone interface [42]. Conversely, applying excessive strain through accelerated loading leads to increased formation of scar tissue, which can adversely affect the tendon-bone interface and alter the mechanical stress at the entrance of the tunnel, thus exacerbating laxity [43, 44]. Packer et al. reported that immediate high-strain loading has a detrimental effect on healing in rat models [44]. Consequently, the authors of this study suggest that rehabilitation protocols allowing full WB in the early stages of recovery should be approached with caution, taking into consideration the different phases of ligament healing depending on the risk factors identified for residual postoperative laxity: high PTS and high pre-operative anterior tibial translation [45]. We did not perform any analysis for bone tunnel enlargement and associated increased laxity in the present study. Nevertheless, current evidence suggests that tunnel enlargement does not significantly impact short-term clinical outcomes. However, it would be premature to conclude that it has no effect on prognosis [46, 47].

Another important aspect highlighted by this study is the impact of PTS. The ACL plays a crucial role in controlling ATT caused by axial tibial forces [48, 49]. The literature consistently recognizes that a higher degree of PTS is associated with increased stress on the ACL [4, 5]. Moreover, the inclination of the tibial surface inherently contributes to tensile forces on the ACL, and predispose the ACLR to fatigue failure [21, 50, 51]. It is well-known that early WB affects the vulnerable tendon-bone interface following ACL reconstruction, particularly in individuals with steeper posterior PTS [52]. However, our study revealed that postoperative SATT increased in WB group who had a smaller PTS than the WB group. This suggests that full WB was the primary factor influencing the difference in pre vs post operative SATT, irrespective of PTS, and was not influenced by choice of fixation, screw vs suspensory.

Finally, the results showed a high incidence of meniscal injuries in patients with PTS ≥ 12° and SATT ≥ 5 mm: this aspect highlights very clearly how a meniscal deficit can impact tibial translation and, consequently, joint laxity [53, 54]. In this regard, the decision to allow full weight-bearing in the postoperative period must take into account any meniscal injuries, even when the meniscus cannot be saved, as its role as a secondary stabilizer is lost, increasing stress on the ACL. In this regard, the sub-analysis conducted in this group did not show statistically significant differences in terms of SATT improvement between patients who did not have meniscal injuries and those who underwent meniscectomies or meniscal repairs. Therefore, the NWB protected their knees from worsening SATT, even those patients for whom the meniscus could not be saved.

As the principle aim of the present study was mainly radiographic assessment, it only reports on the radiographic outcomes and does not correlate these findings directly to clinical outcomes. However, the recent meta-analysis conducted by Fan et al. [12] did not reveal statistically significant differences between the WB and NWB group in terms of clinical scores such as Lysholm, Tegner, and KOOS. Only the subjective IKDC score exhibited higher values in the WB group. Consequently, the superiority of an accelerated protocol over a delayed one remains a contentious aspect.

We acknowledge some limitations. First, it was a retrospective study, and it is subject to the limitations and biases of such studies. Secondly, we did not report clinical outcome scores or the effect of the treatment protocol on re rupture rates. This will be a secondary study, where we will need a minimum of two years of outcomes. Also, this study does not have a direct control group with a similar slope and SATT, however a previous investigation demonstrated that the SATT was not improved following ACLR with a post-operative WB protocol [21]. In this previous investigation, factors associated with a worsening of the SATT were increased tibial slope, medial meniscus injury and high pre-operative SATT, therefore the goal of this study was to investigate the effect of NWB following ACLR in patients deemed at risk due to these factors. Nevertheless, higher-level studies, such as randomized trials, may in the future more accurately assess the role of WB on anterior tibial translation and reinforce the findings of the current study.

## Conclusions

The post-operative protocol of 21 days without WB led to an improvement in SATT at 9 months without an influence on DATT. This post operative protocol is recommended for patients with a SATT ≥ 5 mm, and/or a PTS ≥ 12°, and/or the presence of a root or radial type meniscal lesion as part of an “à la carte” approach to ACL reconstruction. The influence of this protocol on re rupture rates now needs to be evaluated.

## Data Availability

The datasets generated and analysed in the current study are not policy available due to data protection regulations. Access to data is limited to the researchers who have obtained permission for data processing. Further inquiries can be made to the corresponding author.

## Abbreviations

ACL: Anterior cruciate ligament
PTS: Posterior Tibial Slope
SATT: Static Anterior Tibial Translation
DATT: Dynamic Anterior Tibial Translation
WB: Weight bearing
NWB: Non-weight bearing
SD: Standard deviation
SG: Semitendinous-Gracilis
ST4: Quadrupled Semitendinous
